# Regulation of Heme Oxygenase and Its Cross-Talks with Apoptosis and Autophagy under Different Conditions in *Drosophila*

**DOI:** 10.3390/antiox10111716

**Published:** 2021-10-28

**Authors:** Terence Al L. Abaquita, Milena Damulewicz, Debarati Bhattacharya, Elżbieta Pyza

**Affiliations:** Department of Cell Biology and Imaging, Institute of Zoology and Biomedical Research, Jagiellonian University, 30-387 Cracow, Poland; terence.al.abaquita@uj.edu.pl (T.A.L.A.); milena.damulewicz@uj.edu.pl (M.D.); d.bhattacharya@doctoral.uj.edu.pl (D.B.)

**Keywords:** reactive oxygen species (ROS), antioxidant, circadian clock, neuroprotection, aging, paraquat, curcumin, *Drosophila melanogaster*

## Abstract

Heme oxygenase (HO) is one of the cytoprotective enzymes that can mitigate the effects of oxidative stress. Here, we found that the *ho* mRNA level oscillates in the brain of *Drosophila melanogaster* with two minima at the beginning of the day and night. This rhythm was partly masked by light as its pattern changed in constant darkness (DD). It followed a similar trend in the clock mutant *per^01^* under light/dark regime (LD12:12); however, differences between time points were not statistically significant. In older flies (20 days old), the rhythm was vanished; however, 15 days of curcumin feeding restored this rhythm with an elevated *ho* mRNA level at all time points studied. In addition, flies exposed to paraquat had higher *ho* expression in the brain, but only at a specific time of the day which can be a protective response of the brain against stress. These findings suggest that the expression of *ho* in the fly’s brain is regulated by the circadian clock, light, age, exposure to stress, and the presence of exogenous antioxidants. We also found that HO cross-talks with apoptosis and autophagy under different conditions. Induction of neuronal *ho* was accompanied by increased transcription of apoptosis and autophagy-related genes. However, this trend changed after exposure to curcumin and paraquat. Our results suggest that HO is involved in the control of apoptotic and autophagic key processes protecting the brain against oxidative damage.

## 1. Introduction

The brain houses complex groups of neuronal and glial cell types that work collaboratively or independently in coordinating several processes in the organism’s body [1]. This sophisticated function requires continuous high energy supply [2], particularly for synaptic transmission [3], which is correlated with the overproduction of reactive oxygen species (ROS). Excessive accumulation of oxygen free radicals may damage DNA and proteins [4,5,6] that, as a consequence, disturb cellular processes [7]. This condition is regarded as a state of oxidative stress that negatively affects the normal functioning of the brain. This cellular threat is prevalent during aging, as aged brains have been reported to produce high levels of oxidative stress-induced mutations in the mitochondrial DNA [8,9,10,11]. Neurodegenerative disorders are also associated with cellular damage incurred by oxidative stress [12,13,14]. Frequent exposures to adverse conditions, such as radiation, UV light, air pollution, and toxic substances, can also contribute to ROS generation [7].

Neutralizing ROS includes the generation of protective enzymes, e.g., superoxide dismutases [SODs], glutathione peroxidases [GPxs], and catalases [CATs] as well as nonenzymatic endogenous antioxidant molecules such as glutathione (GSH) [15,16,17]. Behavior also plays an important role in regulating the effects of accumulating ROS—not excluding the fact that highly mobile animals have more chances of being exposed to toxic conditions, and sleep has been demonstrated to defend against oxidative challenges [18]. Oxidative stress typically correlates with oxidative damage and cell death. Dysregulated redox signaling and dysfunctional or damaged organelles after stress are removed to maintain cellular homeostasis by the degradation and recycling process called autophagy or elimination of unwanted cells through apoptosis. These processes help neurons in particular to cope with prolonged and sustained operational stress [19]. All these protective measures (generation of antioxidative enzymes, behavior, autophagy, and apoptosis) for redox homeostasis are regulated by the circadian clock, which also controls other cyclic processes [20]. 

Circadian clocks are responsible for generating endogenous oscillations with a period of about 24 h in almost all processes in the body. The molecular mechanism of the clock in *Drosophila melanogaster* depends on the cyclic expression of core clock genes *period* (*per*), *timeless* (*tim*), *Clock (Clk),* and *cycle* (cyc) [21]. CLK/CYC heterodimers act as the transcription factors of *per* and *tim,* which activate their expression at the end of the day as well as other clock genes including *vrille* (*vri*), *Par domain protein 1ε* (*Pdp1ε*), and clock-controlled genes (*ccg*s). During the night, PER and TIM proteins are synthesized and bound to form heterodimers. When PER/TIM heterodimers enter the nucleus, the transcriptional factors CLK/CYC are suppressed by them [22], which inhibits transcription of their own genes. This mechanism of the molecular clock is called the negative feedback loop. In the morning, light activates cryptochrome (CRY) protein, which irreversibly and directly binds to TIM, causing its degradation in proteasomes [23,24]. The dissociation of PER/TIM produces unstable monomeric PER, which is later degraded. The absence of PER/TIM allows for the activation of *Clk* and *cyc* resetting the clock to start the next circadian cycle. Genetic and environmental disruption of the circadian clock can lead to the inability of cells to regulate redox homeostasis [25]. Aging influences the circadian clock as its regulation weakens over time [26]. 

Heme oxygenase (HO) is one of the enzymes under control of the circadian clock both in mammals and in *D. melanogaster* [20,27,28]. HO catalyzes the degradation of heme into carbon monoxide (CO), ferrous ions, and biliverdin. In mammals, HO is present in two isoforms: inducible HO-1 and constitutive HO-2, which are encoded by two different genes. In the suprachiasmatic nuclei (SCN), a site of mammalian circadian pacemaker for behavioral rhythms, HO activity changes during the day, reaching the maximum at night [28]. Furthermore, both proteins scavenge ROS, denoting HO-1 and HO-2 to be cytoprotective and anti-apoptotic agents in neutralizing the effects of oxidative stress [29]. In *D. melanogaster*, HO is encoded by only one gene (*ho*) [30], which shows circadian cycling in the whole head homogenates [20], in the retina and glial cells [27]. However, each of these tissues exhibited differences in the daily expression pattern, which suggests tissue-dependent activity of HO. The protective mechanisms of HO were demonstrated in the retina, where HO plays an important role in the viability, development, iron accumulation, cell death [31], and signaling pathway of DNA damage [32]. It has also been documented that HO defends the retina against oxidative stress in a time-dependent manner. The peak of *ho* expression at the beginning of the day in the retina protects photoreceptors from DNA damage caused by UV and white light [27,33], whereas the second peak at night controls expression of the immune response genes. The circadian cycling of *ho* mRNA also affects the expression of clock genes and other processes present in the retina, such as phototransduction, DNA repair, and autophagy [34]. 

It is hypothesized that *ho* expression also cycles in the brain and protects neurons and glia from oxidative injury. The aim of this study was to examine *ho* expression in the brain, isolated from the rest of the head tissues during the day in young and old flies, and after exposure to oxidative stress or antioxidants. We found that the *ho* gene cycles in the brain in young but not in old flies, and its mRNA level changes after exposure to paraquat (ROS producer) or curcumin (ROS scavenger). In addition, our data suggest that the protective mechanism of HO seems to result from the regulation of apoptosis and autophagy.

## 2. Materials and Methods

### 2.1. Animals

The following strains of *D. melanogaster* were used: wild-type Canton S (CS) strain; *per^01^* (null mutant of the *period* gene)*; elav*-GAL4 (a strain that expresses GAL4 under the control of the *embryonic lethal abnormal vision* (*elav*) promoter; pan-neuronal cell marker); Upstream Activating Sequence or UAS-*ho*RNAi (a strain which expresses dsRNA for *ho* gene under the control of UAS sequence); UAS-*ho* (a strain which expresses *ho* under the control of UAS sequence); UAS-*Valium10* (control for RNAi strains); and *repo-*GAL4 (a strain that expresses GAL4 under the control of the *reverse polarity* (*repo*) promoter; pan-glial cell marker). Flies were maintained under 12 h of light followed by 12 h of darkness (LD12:12) or in constant darkness (DD) at a constant temperature of 24 °C.

### 2.2. Experimental Groups

To detect cyclic expression of *ho* in the brain, 10-days-old CS or *per^01^* males were sacrificed at six time points in LD12:12 or DD conditions. Wild-type (CS) flies, 10-days-old, were used as control groups for the succeeding experiments particularly on aging and paraquat exposure. To study whether aging influences *ho* expression as well as the autophagy-related gene in the brain, 20-days-old CS males were sacrificed at six time points under LD12:12.

To understand the effects of HO on apoptosis, autophagy and DNA repair gene expression, the GAL4/UAS system was exploited to produce flies with overexpressed or silenced *ho* expression in all neurons or in all glia.

#### 2.2.1. Curcumin Treatment

CS males, 5-days-old, were fed with a standard diet ((yeast-cornmeal-agar) supplemented with curcumin at 1 mg/mL of the medium for 15 days. Curcumin (EMD Millipore Corp., Darmstadt, Germany) was dissolved in 1% ethanol and mixed in the standard medium. This concentration has been shown to increase SOD and CAT activity [35]. Control groups were fed with the standard medium with 1% ethanol or with the standard medium only. Longer feeding time (15 days) of curcumin supplementation was implemented as it showed a significant increase in *ho* mRNA level compared to shorter ones. We initially found a strong induction of *ho* expression in the brain at ZT20 and we used this time point in comparing *ho* mRNA levels in the brain at different feeding times (5, 10, and 15 days) of curcumin, which was introduced to 5 days old flies (Supplementary Figure S1).

#### 2.2.2. Paraquat Treatment

CS males, 10-days-old, were fed with a 10 mM concentration of paraquat dichloride (synonymous to methyl viologen dichloride hydrate, Sigma-Aldrich, Steinheim, Germany) mixed in the fly’s standard diet at six different time points for 24 h. Control flies were fed only with the standard culture medium. This exposure time and concentration induced mortality and upregulated SOD activity [36].

### 2.3. RNA Isolation, cDNA Synthesis and Quantitative PCR

Males, 10 or 20 days old, were decapitated at ZT1, ZT4, ZT8, ZT13, ZT16, and ZT20 (ZT Zeitgeber Time, ZT0 indicates lights-on and ZT12 lights-off) in LD12:12 conditions or at CT1, CT4, CT8, CT13, CT16, and CT20 (CT Circadian Time, CT0 and CT12 stand for the beginning of the subjective day and the beginning of the subjective night, respectively) in DD conditions. These time points were selected in our previous study, in which significant differences in the expression of *ho* and many clock-controlled genes in the retina were shown [27,37,38]. Heads were fixed in 100% ethanol for 2 h, and brains were isolated. Approximately 20 flies were used for every time point, and each experiment was repeated at least three times.

Total RNA isolation was performed using TriReagent (MRC Inc., Irvine, CA, USA) according to the manufacturer’s protocol. cDNA was synthesized using a High Capacity cDNA Reverse Transcription Kit (Thermo Fisher Scientific, Vilnus, Lithuania) with random primers according to the provider’s instruction. Gene expression was examined using StepOnePlus Real-Time PCR System and SYBR Green Master Mix (KAPA Biosystems, Cape Town, South Africa) in the presence of primer sequences for the genes used in this study which are listed in Table 1.

Product specificity was assessed by melting curve analysis, and selected samples were run on 1% agarose gels for size assessment. A standard curve was used to calculate gene expression level. The number of target gene copies was normalized to the geometric mean of the *rpl32* gene, previously identified as the housekeeping gene.

### 2.4. Statistical Analysis

Statistical analysis was performed using non-parametric analysis of variance (ANOVA), a Kruskal–Wallis test, and a Conover–Iman’s test to check the rhythm of *ho* expression and a non-parametric Mann–Whitney test to compare between experimental and control groups. All data analyses were performed with the R freeware statistical package version 3.6.2 (http://www.R-project.org/ (accessed on 9 April 2021)) and GraphPad Prism 7.05 software (La Jolla, CA, USA).

## 3. Results

### 3.1. Expression of ho in the Brain under LD12:12 and DD

**Figure 1:**
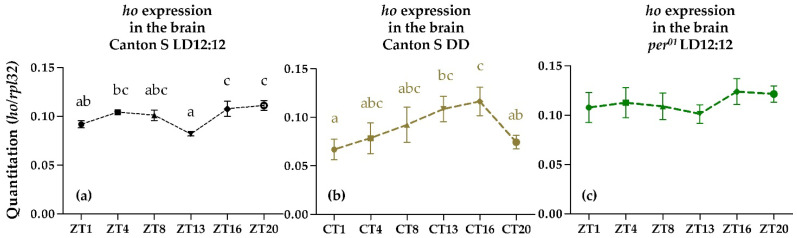
Expression of *heme oxygenase* (*ho*) gene in the brain of male wild-type adult *D. melanogaster* at six time points in LD12:12 (**a**), DD conditions (**b**) and in *per^01^* mutant (**c**) in LD12:12 shown as means ± SD (*n* = 3 repetitions in each time point). Statistically significant differences between time points are indicated with different letters (*p* in LD = 0.0046; *p* in DD = 0.0124; *p* in *per^01^* = 0.2662). LD: 12 h of light and 12 h of darkness; DD: Constant darkness; ZT: Zeitgeber Time; CT: Circadian Time.

The examination of the *ho* mRNA level at six time points during 24 h in LD12:12 conditions showed that *ho* expression cycles in the fly’s brain. The mRNA levels of *ho* in the brain were very low and significant differences were only observed between ZT1 and ZT13 when compared with ZT16 and ZT20 (Figure 1a). This pattern was changed in DD, but the rhythm was maintained (Figure 1b). In addition, the *ho* mRNA level over the course of the day and night was examined in arrhythmic *per^01^* mutant (Figure 1c), which did not result in statistically significant differences between all time points studied. However, the expression pattern of *ho* in *per^01^* mutant had a similar peak and trough with wild-type flies in the same condition (LD12:12), which implies that light can generate a weak exogenous rhythm of *ho* mRNA.

### 3.2. Effects of Aging on ho and Autophagy Gene Expression in the Brain

**Figure 2:**
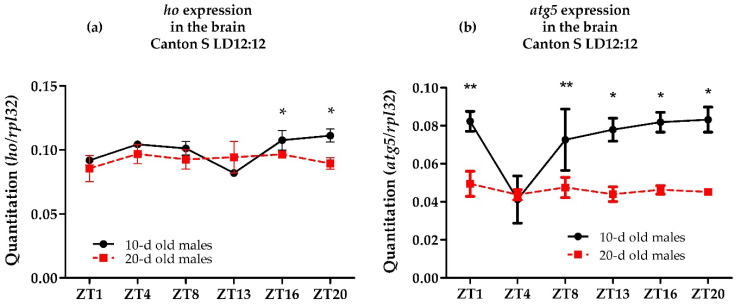
Effects of aging on the daily mRNA expression of *heme oxygenase* (*ho*) (**a**) and *autophagy- related 5* (*atg5*) (**b**) in the brain of adult male *Drosophila melanogaster*. Data shown as means ± SD. Statistically significant differences between groups are indicated with asterisks (10-d old males vs. 20-d old males: (*) *p* < 0.05 or (**) *p* < 0.01 at ZT16 and ZT20 for *ho* expression and ZT1, ZT8, ZT13, ZT16 and ZT20 for *atg5* expression).

The effect of aging on *ho* expression in the brain was examined in 20 days old wild-type flies at six different time points in LD12:12 and compared with younger flies (10 days old) (Figure 2a). We found that *ho* is not cyclically expressed in the brain of older flies, and the mRNA level is decreased in the late evening. Loss of the daily rhythm and significant reduction of mRNA level of *atg5* were also detected in 20 days old flies (Figure 2b). 

### 3.3. Effects of Overexpressing and Silencing of ho on Apoptosis, Autophagy, and DNA Repair

**Figure 3:**
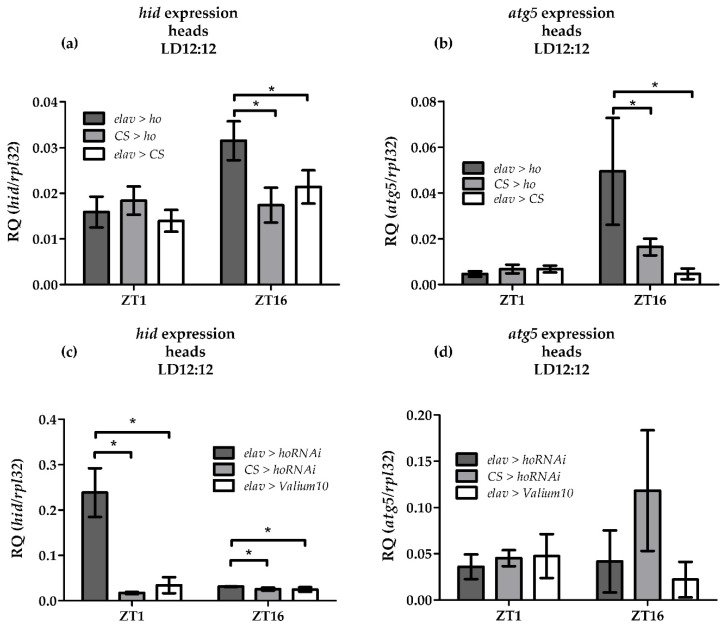
Effects of pan-neuronal overexpression of *heme oxygenase* (*ho*) on the expression of the apoptosis activator *head involution defect* (*hid*) (**a**) and *autophagy-related 5 (atg5*) genes (**b**) mRNA at ZT1 and ZT16 as well as the effects of pan-neuronal silencing of *ho* on the expression of *hid* (**c**) and *atg5* (**d**). Data shown as means ± SD. Statistically significant differences between groups are indicated with asterisks (*elav* > *ho* or *elav* > *hoRNAi* vs. controls: *p* < 0.05).

The differences in daily mRNA levels of the genes encoding apoptosis, autophagy and DNA repair proteins were analyzed between the controls and the flies with pan-neuronal overexpression or silencing of *ho* in *Drosophila* at two different time points (ZT1 and ZT16). The expression of the apoptosis activator gene *head involution defect* (*hid*) significantly increased at night in flies with overexpressed *ho* in all neurons (Figure 3a). This is a time-dependent effect because elevating *ho* transcription did not change *hid* expression in the morning. In addition, silencing of *ho* expression in all neurons also showed a higher level of *hid* mRNA (Figure 3b). This result implies that changes in *ho* expression in neurons can strongly affect apoptosis.

Increasing *ho* expression in all neurons was also accompanied by the induction of *atg5* transcripts at night (Figure 3c). The *atg5* and *hid* mRNA expression seems to be regulated by high transcription of *ho* at night since decreasing *ho* expression did not affect the *atg5* transcript levels (Figure 3d). 

These observations were only detectable in neuronal *ho* as overexpression of *ho* in all glia did not produce significant changes in the mRNA levels of apoptosis and autophagy-related genes. In addition, the protective mechanism of HO in neurons seems to be connected to the regulation of apoptosis as well as with autophagy, and not for DNA repair as mRNA of *eIF4a* (*translation initiation factor* gene) and *Xpc* (*recruits nucleotide excision repair* gene) did not change after overexpressing or silencing *ho* in all neurons and specifically elevating *ho* expression in all glia (Supplementary Figures S2–S5).

### 3.4. Effects of Chronic Curcumin Supplementation on ho, Apoptosis, and Authophagy Gene Expression in the Brain

**Figure 4:**
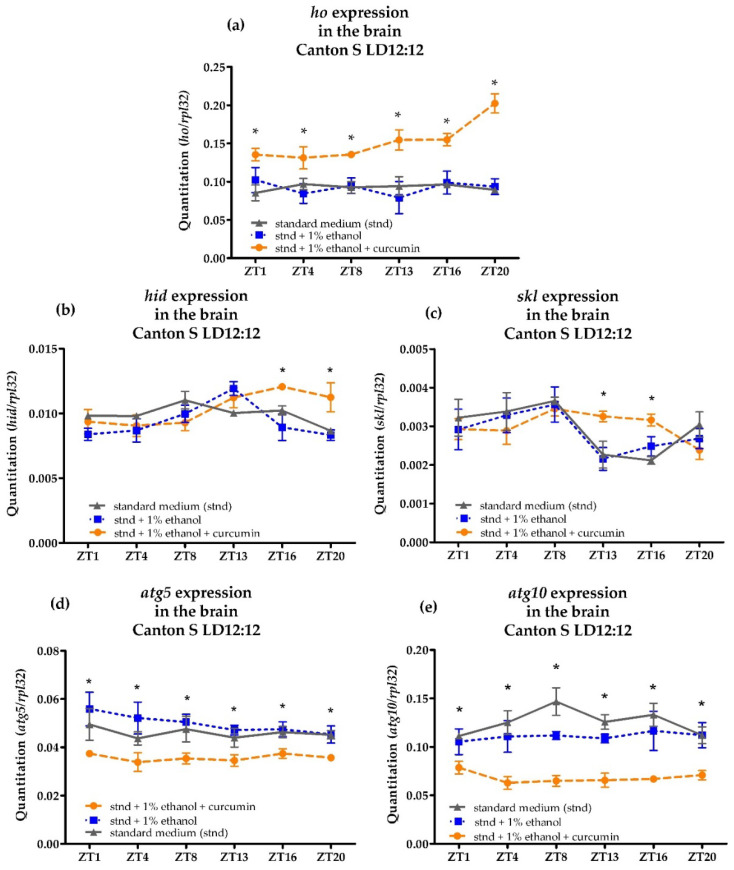
Effects of chronic curcumin supplementation on the mRNA level of *heme oxygenase* (*ho*) (**a**), apoptosis activator *head involution defect* (*hid*) (**b**) and *sickle* (*skl*) (**c**), and *autophagy-related 5* (*atg5*—(**d**) and *atg10*—(**e**) genes in the brain of adult males of *Drosophila melanogaster*. Data shown as means ± SD. Statistically significant differences between groups are indicated with asterisks (*p* < 0.05).

Chronic supplementation of wild-type flies with curcumin strongly induced *ho* expression. We detected a significant increase in the *ho* mRNA level in the brain at all time points studied (Figure 4a). We also found that the supplementation of food with curcumin prevented the loss of rhythm in *ho* expression in the brain of older flies (20 days old). This increase of *ho* mRNA level in the brain after consecutive 15 days of curcumin feeding was accompanied by the induction of *hid* expression at ZT16 and ZT20 (Figure 4b). In the same experiment, we also analyzed the expression of the *skl* gene, another gene coding apoptotic protein, which also showed an increase in mRNA level during the night: ZT13 and ZT16 (Figure 4c). This time-dependent upregulation of apoptotic genes in the brain after long feeding time (15 days) of curcumin, which had a similar effect when the *ho* gene was overexpressed in all neurons, confirmed association of *ho* transcription with apoptosis. However, it was not consistent with *atg5* expression in the brain after chronic curcumin supplementation, since we found significant reduction in *atg5* and *atg10* mRNA levels at all time points studied when *ho* expression was induced by curcumin (Figure 4d,e).

### 3.5. Effects of Acute Paraquat Exposure on per, atg5, Hid and ho mRNA Level in the Brain

**Figure 5:**
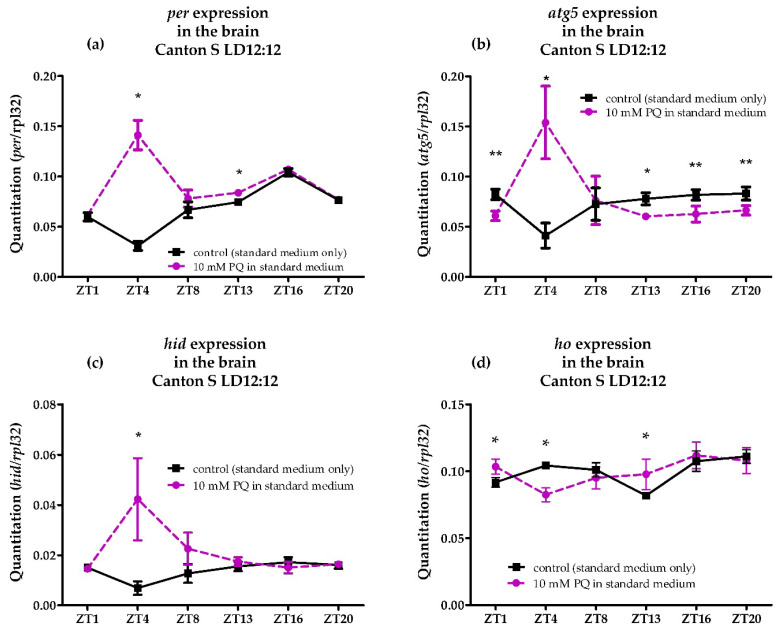
Effects of paraquat (PQ) exposure (for 24 h) on the mRNA level of *period* (*per*) (**a**), *autophagy-related 5* (*atg5*) (**b**), apoptosis activator *head involution defect* (*hid*) (**c**), and *heme oxygenase* (*ho*) (**d**) genes in the brain of adult males of *Drosophila melanogaster*. Data shown as means ± SD. Statistically significant differences between groups are indicated with asterisks (* *p* < 0.05 and ** *p* < 0.01).

Flies were shown to undergo stress after being exposed to paraquat at 10 mM concentration for 24 h. Firstly, we found a disrupted circadian expression pattern of the *per* gene in the fly’s brain with the highest mRNA level in the middle of the day (ZT4) (Figure 5a). We also detected a reverse pattern in the daily oscillation of *atg5* transcripts in the brain, reaching the maximum level at ZT4 (Figure 5b). Paraquat also induced *hid* expression at ZT4 which resulted in a different cycling pattern of *hid* transcription during the day (Figure 5c). The examination of *ho* mRNA level in the brain after acute paraquat exposure showed changes in the daily pattern of *ho* expression compared to the control (Figure 5d). We found a significant increase in *ho* mRNA level in the brain after acute paraquat exposure at ZT1 or ZT13, which in normal condition (LD 12:12) had the lowest mRNA levels. This particular response after acute exposure to paraquat may be explained as a time-dependent protection by *ho* of the fly’s brain during stress. High activity of males at the beginning of the day and night is correlated with higher exposure to toxic insults, and *ho* expression seems to be activated in response to stress. The significant decrease of *ho* mRNA in the brain at ZT4 by paraquat was accompanied by the increase of *per*, *atg5*, and *hid* mRNA.

## 4. Discussion

In the present study, we showed that the gene encoding heme oxygenase (*ho*) in the brain of *Drosophila* is regulated by the circadian clock and by light exposure. The *ho* transcript level in the brain changes during the day and night under LD12:12 conditions, with lowest levels at the beginning of the day (ZT1) and the beginning of the night (ZT13). This rhythm has a similar pattern in *per* null mutants, which indicates that light may generate a weak exogenous rhythm of *ho* mRNA in the brain. In the case of DD, the pattern of *ho* expression was changed, but the rhythm was maintained since the significant difference between CT1 and CT16 was observed. It means that the circadian pattern of *ho* is masked by light in LD12:12. Some clock-controlled genes (*ccg*s) like *Tor, atg5,* and *atg7* in the brain were reported to show differences in the expression pattern in LD12:12 and DD [39], like in the present study in case of *ho* mRNA. The same phenomenon was observed in the rhythm of locomotor activity in *Drosophila* which is bimodal in LD12:12 but unimodal in DD.

In the mammalian brain, in which HO-1 was also detected, its concentration was very low [40,41,42] in contrast to the retina, which is exposed to intense UV and visible light-induced oxidative stress [43]. Using the publicly available visualization tool for large-scale single cell RNA sequencing (scRNA) analysis, known as SCope (http://scope.aertslab.org/ (accessed on 15 October 2020)), we verified the presence and distribution of *ho* mRNA in the brain. The *ho* gene was detected at various expression levels in different cell clusters in the fly’s brain, with the highest expression in the optic lobe, followed by the expression in the central brain and then in glial cells. Higher expression of *ho* in the optic lobe is expected since the visual system is especially affected by oxidative stress as a result of the phototransduction [44]. 

It has already been found that *ho* is a clock-controlled gene (*ccg*) in the retina [20,27]. The brain is a heterogeneous tissue composed of different cell clusters which have different expression patterns of the *ho* gene (SCope). This may be the reason why the pattern of *ho* expression was not the same in the brain as in the retina. Our study also showed that the daily oscillation of *ho* transcription in the brain differs in the daily pattern compared with whole head homogenates which included retinas [20]. However, the rhythm observed in the *Drosophila* brain was similar to that detected in isolated glial cells under light/dark regime in our previous study [27]. The expression pattern of *ho* in glia was strongly rhythmic; however, it was not examined in DD or in *per* null mutants to verify if this rhythm was clock-controlled.

Aside from light, the transcription of *ho* in the brain is influenced by other factors which we documented in our study. Firstly, we found that aging affects HO because the rhythm of *ho* mRNA level in the brain in younger flies was abolished in older flies. We also detected a decline in the *ho* mRNA level in the brain of older flies, which is a common response in the aging brain since the total RNA level decreases overtime [45]. A decrease of *ho* mRNA cycling during aging has also been observed in mammals [42,46]. This can be explained by the fact that aging is associated with weakened circadian oscillations [26] that negatively affect clock-dependent processes [47]. Secondly, we observed that curcumin supplementation, an antioxidative compound, leads to upregulation of *ho* in the brain, regardless at which time of the day it was examined. A similar observation was made in the case of HO-1 since its mRNA and protein levels increased after exposure to curcumin [48,49,50,51,52,53,54,55], which can result from activating the nuclear factor, erythroid 2 like 2 (Nrf2) pathway [54,55]. Nrf2 and its inhibitor Kelch-like ECH associated protein (Keap) 1 regulate the expression of several cytoprotective genes [7]. HO-1 is known as a Nrf2-dependent gene and, when Nrf2 siRNA was used, curcumin-induced HO-1 expression was significantly reduced [54]. 

Finally, we showed that, under stress, mediated by the ROS inducer paraquat, *ho* expression in the brain was increased. The same result was observed in mammals in which HO-1 mRNA was induced after the exposure to paraquat [56]. The HO-1 gene was previously reported to be regulated by several factors, including curcumin, paraquat, or aging; however, it has not been studied in *Drosophila* and similar effects have not been expected. Moreover, we focused on HO at the transcription level since mRNAs are more sensitive to internal and external factors than proteins.

The specific expression of HO during the day in the fly’s retina was reported to control several processes, including neuroprotection and the innate immune system. Here, we found that neuronal HO cross-talks with apoptosis and autophagy in a time-dependent manner. Apoptosis is a process of programmed cell death which eliminates unwanted or damaged cells, while autophagy is a cellular degradation and recycling process for cytosolic macromolecules and damaged organelles. Both processes work to maintain cellular homeostasis and are intimately associated with each other. Apoptotic signaling can regulate autophagy and conversely autophagy can regulate apoptosis (not exclusive to other cell death mechanisms) [57]. Notwithstanding, molecular interactions between autophagy and cell death are complicated and may have different contexts. However, it is possible that the same proteins can control both apoptosis and autophagy [57], just like we found in our study in case of HO. The interactions between apoptosis, autophagy, and HO have already been reported in mammals depending on injury. In *Drosophila*, apoptosis and autophagy genes were downregulated after increasing *ho* expression [34]. We found, however, an opposite effect when we genetically induced *ho* expression only in all neurons. The apoptosis activator *head involution defect* (*hid*) and autophagy gene *atg5* expression were increased at night in whole head homogenates when the *ho* gene was overexpressed. However, when we silenced *ho* in neurons, *hid* expression increased but *atg5* remained at the same level as in the control. This implies that any changes of the physiological level of neuronal HO can trigger apoptosis. An association of neuronal HO with autophagy was shown to enhance *atg5* transcription when the *ho* mRNA level was high during the night. These differences may be attributed to the fact that our samples, which we used for analyses of apoptosis or autophagy transcript levels, were from whole heads and not from specific head tissues. There might be additional or compensation effects from other cells of the head after silencing or overexpressing *ho*. Nevertheless, we did not find any connections of autophagy and apoptosis with glial HO. In addition, we hypothesize that *ho* expression affects DNA damage repair genes as HO was shown to reduce DNA damage in the retina during phototransduction and/or UV radiation [27,33]. Surprisingly, we did not observe any changes in the expression of genes involved in DNA repair after silencing or overexpressing neuronal *ho* or overexpressing glial *ho*. It means that DNA breaks after oxidative stress induce apoptosis activated by HO rather than DNA repair.

A growing body of evidence shows the role of the *Drosophila ho* in oxidative challenges, particularly in the retina [27,31,32,33,34]. We also found that aging may limit the protection by HO. Aging and age-related diseases are a consequence of free radical-induced damages of cellular macromolecules and inability to counterbalance these changes by endogenous antioxidant defense processes, which decline in aged brains. It has been found that, during aging, ROS level increases, along with lipid peroxidation, neurodegeneration, loss of synapses, and memory retention, while SOD, CAT, GSH, and choline acetyltransferase (ChAT) levels decrease [58]. We observed that chronic supplementation with curcumin has a potential to enhance the antioxidant mechanisms in the brain in the early phase of aging. We found the induction of *ho* expression at all time points studied as well as the restoration of the daily rhythm of *ho* mRNA lost in older flies after chronic curcumin supplementation. Furthermore, shorter feeding time with curcumin did not produce a significant increase of *ho* mRNA at the time when the expression level was observed at its peak (ZT20) after 15 days of feeding with curcumin. We assume that curcumin activates neuroprotective processes dependent on HO. This model of using curcumin as an antioxidant and anti-aging compound in mammals has been extensively exploited in neuroprotection by enhancing the HO-1 related pathways [49]. This is supported by the findings about curcumin protection which depends on HO-1 against H_2_O_2_-mediated cell death, and likely through the generation of CO [52]. Anti-apoptotic function of curcumin required the upregulation of HO-1 protein through the PI3K/Akt signaling pathway [53]. However, there is a need to further verify the extent to which the induction of *ho* expression after curcumin treatment protects the brain from aging or oxidative stress. We have only checked daily oscillations of *ho* mRNA levels with the chronic supplementation of curcumin under normal conditions and not combined with any stress factors. Nevertheless, curcumin also induces endoplasmic reticulum stress causing calcium release and destabilization of the mitochondrial compartment, which results in apoptosis. Autophagy in this case failed to rescue all cells and most cells underwent cell death [59,60]. We found the same relationship of apoptosis and autophagy expression in the fly’s brain after curcumin feeding. Curcumin induced *ho* expression at all time points studied which was accompanied by the reduction of *atg5* and *atg10* expression, while *hid* and *skl* mRNA levels increased only at night. It has also been reported that inducing autophagy inhibits curcumin-induced cell death [61]. The neuroprotective effects of curcumin can also be linked through modulating various stages of the autophagy signaling pathway [62,63]. Curcumin, through the modulation of *Atg7* and *p62*, can inhibit differentiation, promote cell survival and inhibit cell cycle progression from G1 to S [62]. Our study was only limited to the mRNA expression of autophagy-related genes *atg5* and *atg10*. 

We also propose that HO may play a neuroprotective role against paraquat toxicity in a time-dependent manner. The increase of *ho* mRNA level in the brain 1 h after lights-on and 1 h after lights-off, which we observed, may imply the neuroprotective response under stress and may be linked with the *Drosophila* circadian rhythm in behavior. Males are more active in locomotion at the beginning of the day and night and are therefore more exposed to toxic insults. The expression of *ho* can be increased to defend against stress during these times. However, it was suggested that the induction of HO-1 does not affect paraquat toxicity [64]. Nonetheless, a lesser concentration (50–250 μM) of paraquat was used, and it may be possible that the cells were not stressed at all. The survival after paraquat exposure was higher in flies treated with 5 mM paraquat or lower, and they also survived longer (in days) than those fed with higher concentration (10 mM) of paraquat [65]. The concentration which we used (at 10 mM) was shown to be ingested and lethal after administration in the fly’s standard food. It also upregulated SOD, an enzyme that breaks down superoxide anions which are harmful ROS [36]. 

We also observed a change in the expression pattern of the *period* gene (*per*), along with *ho* transcription, after exposure to paraquat, which confirmed that, in this concentration, flies were under stress. Disrupted *ho* expression due to oxidative stress may also disturb the circadian clock, or vice versa. The effect of HO, mediated by carbon monoxide (CO), was documented to regulate the circadian clock gene expression [27]. Silencing *ho* in the retina increases and decreases the expression of the canonical clock genes *period* (*per*) and *Clock* (*Clk*), respectively, which, as it has been shown, has an opposite effect after increasing *ho* expression [27]. Clock disruption can negatively affect many cellular processes, including oxidative stress [66]. Paraquat also affected the daily expression of autophagy and apoptosis genes in the brain, which can also be linked to the effect of paraquat on HO. At the time when *ho* mRNA level was significantly reduced after acute paraquat exposure, we found accumulation of *atg5* mRNA in the middle of the day, along with the induction of the apoptosis activator *hid* transcript levels. For the rest of the day, autophagy gene mRNA was kept at minimum levels by paraquat which was accompanied by insignificant change in the *hid* expression. This finding confirmed the studies that showed the importance of autophagy in regulating paraquat-induced apoptosis [67,68]. Induction of *ho* by paraquat at the beginning of the day and night may also play a role in stabilizing apoptosis at the required level.

## 5. Conclusions

Our study showed the expression of the gene encoding HO in the fly’s brain cycles during the day and night. This rhythm is generated by the circadian clock but also regulated by light. During aging, HO shows a decline in its neuroprotective function along with autophagy, as mRNA levels of both genes decreased over time and their rhythmic expression patterns were lost in older flies. The loss of rhythmicity in *ho* expression was prevented by curcumin, and there was a significant increase in the *ho* mRNA level at all time points. The results obtained in the present study provide evidence that HO in the fly’s brain plays an important role in cell survival and protects against paraquat-induced oxidative stress. HO can also be enhanced by curcumin to fight age-related oxidative challenges. Neuronal *ho* transcription also regulates apoptosis and autophagy under different conditions, which seems to be its protective mechanism in redox homeostasis. The timing, age, exposure to stress, and the presence of antioxidative factors must be considered to fully optimize the neuroprotective function of HO for therapeutic purposes.

## Figures and Tables

**Table 1:** Primer sequences of the genes which were used in this study.

Gene		Sequence	Accession Number
*ho*	F:	5′-ACCATTTGCCCGCCGGGATG-3′	CG14716
	R:	5′-AGTGCGACGGCCAGCTTCCT-3′	
*rpl32*	F:	5′-TATGCTAAGCTGTCGCACAAATG-3′	CG7939
	R:	5′-AGCACGTGTATAAAAAGTGCCA-3′	
*hid*	F:	5′-CATCCATGGCCACATCAGT-3′	CG5123
	R:	5′-TTACACGTCTCCTGCGCTTT-3′	
*skl*	F:	5′-ACGACAACTCGCCAAGAGTTCAGA-3′	CG13701
	R:	5′-TATCGACTTGATCGCCACTGGGTT-3′	
*atg5*	F:	5′-GACATGCTCGTCAAGCTCAA-3′	CG1643
	R:	5′-TCCATTAGCCTCCGATTGAC-3′	
*atg10*	F:	5′-TCAGACCCTTTATGGCATTG-3′	CG12821
	R:	5′-GGCTTTCCGAAACTGCTTTAG-3′	
*per*	F:	5′-AAGAGCACCTTCTGCGTGAT-3′	CG2647
	R:	5′-AGAATCTCGTCGGGAACCTT-3′	
*eIF4a*	F:	5′-AGCACGTGTATAAAAAGTGCCA-3′	CG9075
	R:	5′-TTGTCGTACACCTCGTGCC-3′	
*Xpc*	F:	5′-AACGTGAAGGGAATCAGCGT-3′	CG8153
	R:	5′-TTTTCGCCTACCTGCCACTT-3′	

## Data Availability

The data presented in this study are available on request from the corresponding author. The data are not publicly available due to the fact that our results are only qPCR data that do not need to be stored in a database.

## Supplementary Materials

The following are available online at https://www.mdpi.com/article/10.3390/antiox10111716/s1, Figure S1: Heme oxygenase (*ho*) expression in the brain of adult male *Drosophila melanogaster* fed with curcumin for 5, 10, and 15 days at ZT20, Figure S2: Effects of pan-neuronal overexpression of heme oxygenase (*ho*) on the expression of the nucleotide excision repair gene *Xpc* at ZT1 and ZT16 (**a**) as well as effects of pan-neuronal silencing of *ho* on *Xpc* expression at ZT1 and ZT16 (**b**), Figure S3: Effects of pan-neuronal overexpression and silencing of heme oxygenase (*ho*) on the expression of the translation factor *eIF4a* at ZT16, Figure S4: Effects of pan-glial overexpression of heme oxygenase (*ho*) on the expression of: *hid* (**a**), *atg5* (**b**), and *Xpc* (**c**) at ZT1 and ZT16, Figure S5: Effects of pan-glial overexpression of heme oxygenase (*ho*) on the expression of the translation factor *eIF4a* at ZT16.
