# Supplementary material for: Regulation of Heme Oxygenase and Its Cross-Talks with Apoptosis and Autophagy under Different Conditions in Drosophila
Source: Antioxidants (Basel). 2021 Oct 28;10(11):1716. doi: 10.3390/antiox10111716 (PMC8614956; doi:10.3390/antiox10111716)
Supplement: Supplementary file 1 [file antioxidants-10-01716-s001.zip › antioxidants-1379296-supplementary.pdf]

antioxidants

MDPI

Supplementary Figures

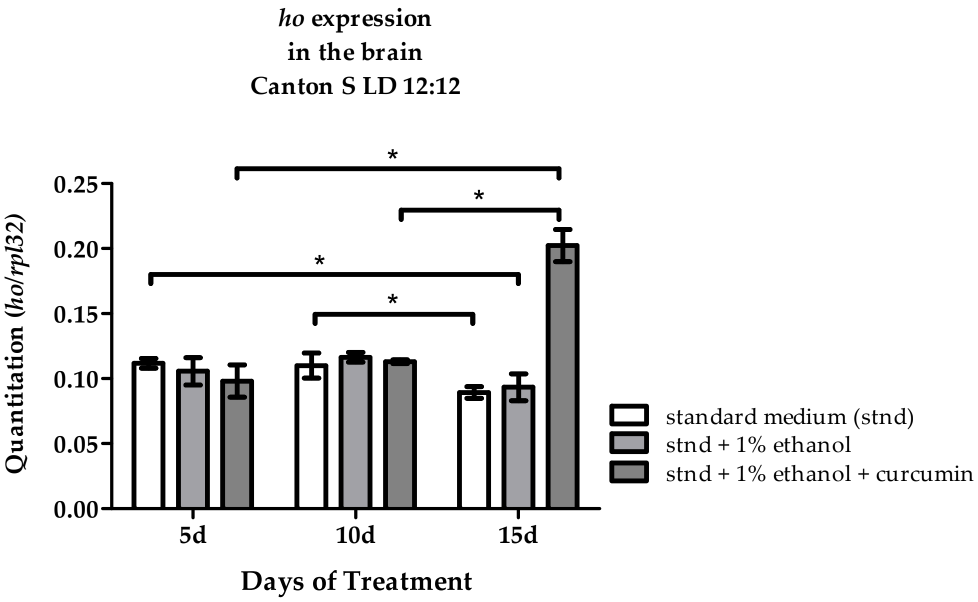

Figure S1. Heme oxygenase (*ho*) expression in the brain of adult male *Drosophila melanogaster* fed with curcumin for 5, 10, and 15 days at ZT20. Data shown as means ± SD. A standard curve was used to calculate gene expression level. Statistically significant differences between groups are indicated with asterisks ($p < 0.05$).

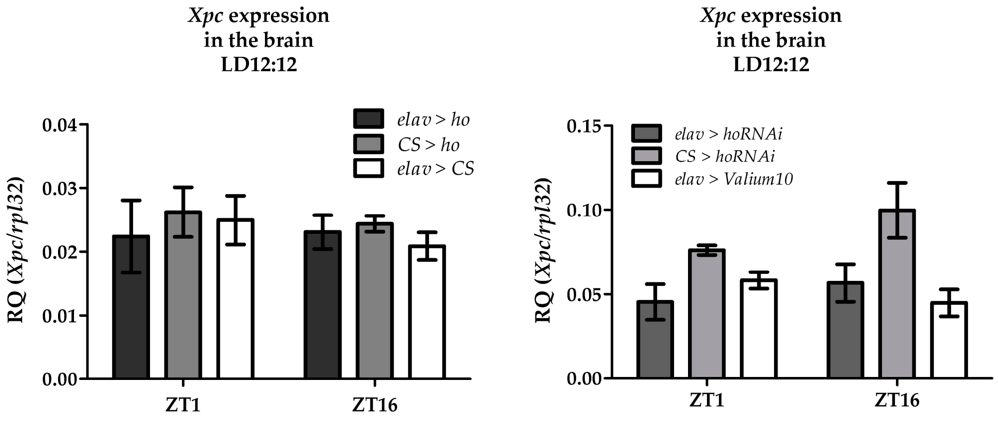

Figure S2. Effects of pan-neuronal overexpression of heme oxygenase (*ho*) on the expression of the nucleotide excision repair gene *Xpc* at ZT1 and ZT16 (a) as well as effects of pan-neuronal silencing of *ho* on *Xpc* expression at ZT1 and ZT16 (b). Data shown as means ± SD. A standard curve was used to calculate gene expression level. Statistically significant differences between groups are indicated with asterisks ($p < 0.05$).

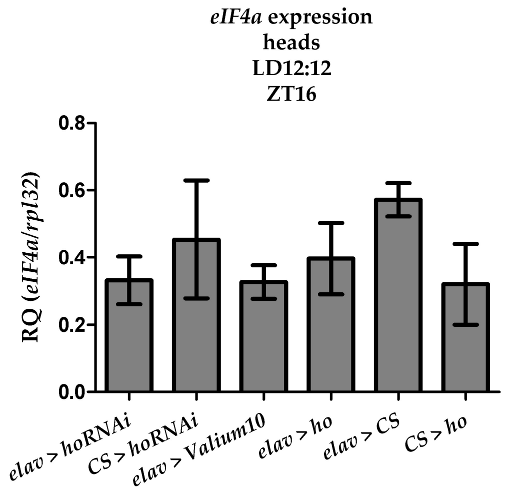

Figure S3. Effects of pan-neuronal overexpression and silencing of heme oxygenase (*ho*) on the expression of the translation factor *eIF4a* at ZT16. Data shown as means ± SD. A standard curve was used to calculate gene expression level. Statistically significant differences between groups are indicated with asterisks ($p < 0.05$).

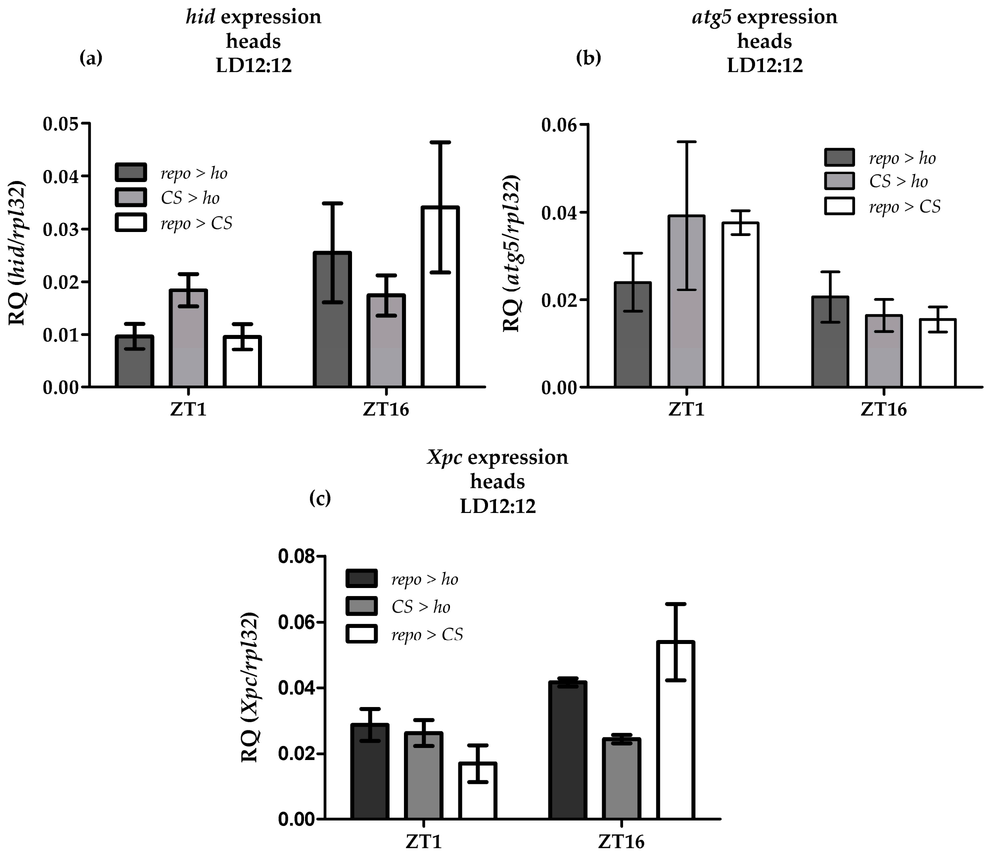

Figure S4. Effects of pan-glial overexpression of heme oxygenase (*ho*) on the expression of: (a) *hid*, (b) *atg5*, and (c) *Xpc* at ZT1 and ZT16. Data shown as means ± SD. A standard curve was used to calculate gene expression level. Statistically significant differences between groups are indicated with asterisks ($p < 0.05$).

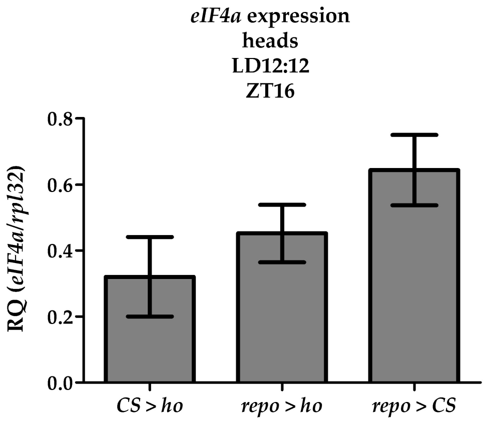

Figure S5. Effects of pan-glial overexpression of heme oxygenase (*ho*) on the expression of the translation factor *eIF4a* at ZT16. Data shown as means ± SD. A standard curve was used to calculate gene expression level. Statistically significant differences between groups are indicated with asterisks ($p < 0.05$).
